# Digital interventions in mental health: An overview and future perspectives^☆^

**DOI:** 10.1016/j.invent.2025.100824

**Published:** 2025-04-02

**Authors:** Johanna Löchner, Per Carlbring, Björn Schuller, John Torous, Lasse Bosse Sander

**Affiliations:** a Department of Clinical Psychology and Psychotherapy, Friedrich-Alexander-University Erlangen-Nuremberg, Germany; b German Center for Mental Health (DZPG), Site Tübingen, Germany; c Department of Psychology, Stockholm University, SE-106 91 Stockholm, Sweden; d School of Psychology, Korea University, Seoul, South Korea; e CHI, Department of Clinical Medicine, Technical University of Munich, Munich, Germany; f GLAM, Department of Computing, Imperial College London, England, United Kingdom; g Department of Psychiatry, Rabb-2, Beth Israel Deaconess Medical Center, MA, United States of America; h Medical Psychology and Medical Sociology, Faculty of Medicine, University of Freiburg, Baden-Württemberg, Germany

**Keywords:** Digital mental health, Treatment and prevention, Implementation, Artificial intelligence, Future perspective

## Abstract

As e-health offerings rapidly expand, they are transforming and challenging traditional mental health care systems globally, presenting both promising opportunities and significant risks. This article critically examines the potential and pitfalls of integrating digital technologies into mental health care, particularly in the realms of diagnosis, prevention, and treatment. It explores current advancements and evidence-based practices, and provides a vision for how future technologies can evolve responsibly to meet mental health needs. The article concludes with the TEQUILA framework, addressing essential elements and challenges for fostering a beneficial and ethical future. A responsible future for digital mental health requires building *Trust* by ensuring data privacy, security, and transparency in AI-driven decisions, along with *Evidence-based* and robust regulatory oversight to maintain *Quality*. *Usability*, design, usability tailored to diverse needs, and ethical alignment with users' *Interests* will all be essential, while *Liability* and *Accreditation* standards will safeguard accountability in this evolving landscape.

## Highlights

• Digital interventions reshape mental health care with both promise and challenges.
• AI, wearables, and VR expand diagnostic and treatment possibilities in psychiatry.
• Ethical concerns include data privacy, transparency, and regulatory oversight.
• The TEQUILA framework outlines key factors for responsible digital mental health.

## 1 Introduction

In a world permeated by digital innovations, we are experiencing an epochal change in almost all areas of life. ChatGPT is one of the fastest-growing services in history despite its nascency. For the upcoming generation, a world without smartphones, online search engines, messenger services, and an (almost) always-available internet connection is no longer imaginable. Indeed, the majority of teens already report being online “all the time” (Santos and Reeve, 2020). For most people, their mobile phone is the first and last thing they use during the day. Even though most children do not have their own smartphone until the age of 10 years, digital media (voice assistants, tablets, smartwatches, etc.) are already taken for granted, leading to confident use, access to various media, and a strong affinity for technology among children and young people from an early age (Chang et al., 2018; Kwok et al., 2017; Feierabend et al., 2021).

Digital media characterise our lives together in many ways: they promote global networking, enable unprecedented access to knowledge and educational materials, e.g., via digital platforms such as e-learning tools, and facilitate instant interpersonal communication.

Considering the field of mental healthcare, telemedicine and health apps promise improved access to care, which seems particularly valuable given the persistently high prevalence rates and ongoing mental health crisis around the globe (Keuroghlian et al., 2023; O'brien and McNicholas, 2020). The greatest hope is probably to improve access to psychiatric and psychotherapeutic care and, especially in regions with limited services or for young people who have difficulties, to intervene early and at a low threshold before crises escalate and become chronic, putting a permanent strain on the system (McGorry et al., 2024). Ideally, the prevention, diagnosis, and treatment of mental illness is flexible and cost-effective (e.g., through scalable mobile applications), culturally and linguistically adapted (Spanhel et al., 2021), needs-, age- and gender-orientated, and accessible independent of the respective location (Ebert et al., 2018; Paganini et al., 2018; Robinson et al., 2016). Vulnerable groups in particular, who often “fall through the cracks” (Bauer, 2005), require targeted interventions that leverage digital media.

Accordingly, the market for telemedicine and health apps is currently experiencing an explosive upswing: driven by the increasing demand for flexible healthcare solutions and intensified by the coronavirus pandemic, the availability of advanced technologies and telemedicine is growing rapidly (Linardon et al., 2019). Forecasts indicate that this market will continue to flourish in the coming years, supported by innovative solutions such as video consultations, remote monitoring, and medical apps (Mikulic, 2022). In 2022, more than 50,000 mHealth offers were available for download in the App Store and Google Play Store (Mikulic, 2022). Most of these offerings relate to psychosocial well-being and stress management, with only 2.08 % of these publicly available mobile apps having published evidence of effectiveness (Baxter et al., 2020; Lau et al., 2020). By 2025, the global mobile health app market is expected to increase from USD 2.4 billion (2017) to USD 11.2 billion (statista.com).

However, while this digital push has the potential to improve care, it also presents challenges that need to be carefully considered (Smith et al., 2023). Data protection, ethics, possible negative effects (Rozental et al., 2018), and the quality of digital interventions are key aspects that require particular attention in psychotherapy practice. In this area of tension between progress and responsibility, the question of how the digital transformation will shape the work of mental healthcare arises.

A critical discussion of the opportunities and risks of using digital technology in the diagnosis, prevention, and treatment of mental illness requires a sound basis on current developments in of digital mental health, the opportunities for treating people and, at the same time, the ethical and practical challenges that arise for professionals. In this article, we focus on current developments, evidence, implementation and recommendation for the successful integration of digital technology into treatment in the first part. In the second part, we create a vision on two narratives on how the future ehealth technologies in mental health care may develop in a responsible and required helpful way. We finalise this article with the TEQUILA-acronym, tackling necessities and challenges to create a beneficial future of digital health technologies.

## 2 Current eHealth development

### 2.1 Technology in the field of mental health treatment

Despite its growing application, particularly in telemedicine and online therapy, research remains limited (Andersson et al., 2019a), with initial studies predominantly focusing on adults (Keuroghlian et al., 2023; O'brien and McNicholas, 2020). The use of digital interventions in the form of so-called Digital Mental Health Intervention (DHI, translation of psychosocial interventions into digital formats) is also no longer a novelty and is integrated into therapy, supported by therapists or offered as self-help tools (Borghouts et al., 2021). In this context, Germany has pioneered the integration of digital health applications (DiGAs) into its healthcare system, with 64 low-risk medical devices approved for use since September 2020, available through statutory health insurers following a prescription or approval (*Bundesinstitut fu¨r Arzneimittel und Medizinprodukte (BfArM): DiGAVerzeichnis*, 2022).

Wearables, such as smartwatches, rings, and bracelets, are less common in health care, but are frequently used on the open market. These devices generate highresolution and longitudinal behavioural, environmental, and physiological data, e.g., sensors on the mattress can detect movement patterns of the chest and derive sleep and respiratory rate data (Bidargaddi et al., 2017). Devices worn on the wrist contain accelerometers, WiFi, a global positioning system, and optical sensors to determine activities, locations, and physiological measurements such as heart rate, sleep patterns, oxygen saturation, and skin temperature. Smartwatches in particular are an increasingly popular tool for primary school children, who can communicate with other children and their parents via voice messages and provide parents with information about their whereabouts via GPS tracking and geo-fencing (emitting a signal when entering or leaving a defined area).

Virtual or augmented reality (VR/AR), the computer generated three-dimensional (3D) simulation of a room or environment (VR) or augmentation of existing ones (AR), is also increasingly being used in the treatment of mental illness (Ma et al., 2021). This technology not only facilitates widespread dissemination of psychotherapy due to its cost-effectiveness but is also preferred by many users over traditional methods, with studies showing a high preference for VR exposure therapy compared to in vivo exposure (Carl et al., 2019). The current standard VR systems use VR headsets with a head-mounted display and offer not only visual and auditory stimuli, but also tactile and olfactory stimuli, for example, to create a series of images and sounds of real situations that can be interacted with in a seemingly realistic way using special electronic devices (Emmelkamp and Meyerbröker, 2021). Areas of application include phobias (Miloff et al., 2019), social anxiety disorder (SAD), depression (Lindner et al., 2019), PTSD, obsessive-compulsive disorder, substance-related disorders, eating disorders, psychosis, and ADHD (Emmelkamp and Meyerbröker, 2021)).

A final area that is becoming increasingly popular refers to chatbots. Also known as conversational agents or voice assistants, these are digital tools that can conduct conversations in natural language and mimic human-like behaviour in task-oriented dialogue with humans (Vaidyam et al., 2021). It is estimated that 42 % of adults in the US use digital voice assistants on their smartphones and 24 % own at least one smart speaker device. However, use in the healthcare sector is (still) low (see reviews by (Abd-Alrazaq et al., 2019; Laranjo et al., 2018). The areas of application mostly related to autism spectrum disorders and depression (Abd-Alrazaq et al., 2019).

A prominent advantage and danger of digital technologies is the large amount of data (big data) collected from patients. This data can be analysed with the help of AI and used for diagnostics, therapy recommendations, and decisions. For example, smart devices such as a smartphone or smartwatch can be used to collect both active data (questionnaires) and passive data (heart rate variability, app usage, GPS location, etc.) in real time. These can be analysed to predict behavioural changes without common sources of error such as memory bias, social desirability (Ghosh et al., 2022; Baumeister et al., 2023; Seizer et al., 2024). As with any other application of a method, the consideration of patient rights under the GDPR (General Data Protection Regulation) is decisive (at least within the European Union)and therefore not a new finding. It becomes problematic when many private companies are less than transparent and methodologically thorough in their processing of personal data, e.g., for commercial and unintended purposes (Larsen et al., 2019). Recent rulings by the Federal Trade Commission in the United States highlight a series of misuse of patient data but also the watchful eye of regulatory bodies now more alert for maleficence (US FTC, 2024a; US FTC, 2024b).

Despite the requirement to present the General Terms and Conditions in an easily understandable and transparent manner, there is a problem with implementation (Andersson et al., 2019b). The so-called privacy paradox refers to the phenomenon in which users who are more likely to disclose their data freely if there is a great subjective benefit, although they would rather refuse to do so without this benefit (e.g., in social networks) (Rössler et al., 2022). In addition, there is an imbalance of privately and freely used DHIs that do not comply with the GDPR guidelines, while evidence-based DHIs must ensure this. Attempts to keep up with the rapid technological development on the research side as well as on the legal side are therefore subject to an unequal race.

### 2.2 Evaluation status

Despite the small number of scientific studies in relation to the range of DHIs (Baxter et al., 2020; Lau et al., 2020; Larsen et al., 2019), the scientific evidence base is growing: in recent years, various meta-analyses have been conducted, particularly of iCBT (internet-based cognitive behavioural therapy) and self-help interventions, which have shown the equivalence of face-to-face therapies (Andersson et al., 2014; Hedman-Lagerlöf et al., 2023; Cuijpers et al., 2019; Hennemann et al., 2018). DHIs have also been confirmed as effective digital assistants in the treatment of mental disorders in meta-analytical studies (Moshe et al., 2021; Firth et al., 2017). Interventions that include a face-to-face element with a professional, peer, or parent have been associated with greater efficacy, higher compliance, and lower dropout rates than self-administered interventions (Lehtimaki et al., 2021; Wright et al., 2019). Increased results on the cost-effectiveness of digital interventions indicate a generally favourable effect in terms of costs and health outcomes (Gentili et al., 2022). However, many DHI studies lack an appropriate control or placebo group, which makes final judgments about effectiveness let alone efficacy or cost efficacy challenging (Goldberg et al., 2023). In addition to treatment, DHIs hold immense potential for the prevention of mental disorders, especially in the form of early interventions for people with subclinical symptoms (indicated prevention) or for people carrying certain risk factors like chronic physical conditions (selective prevention). Several large scaled RCTs have shown the potential of DHI for the prevention of depression in adults. In meta-analytical comparisons, both iCBT (Vigerland et al., 2016; Wu et al., 2023) and DHIs have also been shown to be effective in the treatment of depression and anxiety disorders in children and adolescents compared to waiting control groups, e.g., in the area of anxiety and depressive disorders (Eilert et al., 2022) or behavioural disorders (Florean et al., 2020), albeit with a greatly reduced number of included studies (k ¡ 23 each).

For youth population, existing quantitative studies have primarily investigated either the acceptance or feasibility of mHealth applications (Grist et al., 2017). However, growing evidence supports efficacy of treatment (Wu et al., 2023; Eilert et al., 2022; Florean et al., 2020; Domhardt et al., 2018) and prevention in this group with small effect sizes (Champion et al., 2023; Fusar-Poli et al., 2021; Mens et al., 2022; Newbold et al., 2020; Watkins et al., 2024). Also, there is emerging evidence that guided internet-based psychodynamic therapy could be promising for adolescents with depression (Mechler et al., 2022).

Lindqvist et al. (2024) highlight that the absence of eye contact can be perceived positively, as it enhances a sense of safety and openness, facilitating deeper self-exploration and emotional expression in the therapeutic context (Lindqvist et al., 2024). The use of VR/AR has shown effects for the diagnosis and treatment of people with psychosis (Lan et al., 2023), autism spectrum disorders, and attention deficit hyperactivity disorder and initial evidence of effectiveness for addiction and eating disorders, but little evidence for generalised anxiety disorder and obsessive-compulsive disorder (Emmelkamp and Meyerbröker, 2021). Chatbots with unrestricted natural language input options for health-related purposes are an emerging field of research (Laranjo et al., 2018), showing first promising results regarding the reduction of depressive symptoms and psychological stress (Li et al., 2023). In addition, these studies usually do not yet exploit current possibilities of natural and free language provided by large language models and generative AI. This is largely due to the limited controllability of the output stemming, e.g., “hallucination” and potential data biases in the training of such approaches coming with limited liability, fairness, and explainability due to sheer model size.

Overall, research into the impact and use of telemedicine and DHIs requires further development and is characterised by various limitations: In addition to the small number of studies compared to the supply of DHIs, hardly any samples with psychosocial stress and low-resource environments were included (Lehtimaki et al., 2021). In addition, it should be noted very critically that suicidality is often an exclusion criterion, as ensuring patient safety requires higher resources and monitoring (Sander et al., 2020; Büscher et al., 2022). Further limitations are only short (¡ 12 weeks) or no follow-up measurement points and high drop-out rates of participants, especially in self-help DHIs with up to 75 % (Lecomte et al., 2020; Linardon and Fuller-Tyszkiewicz, 2020). Kolominsky-Rabas et al., (Kolominsky-Rabas et al., 2022) also criticised the evidence situation in the German DiGA approval process as problematic overall and, in addition to the poor study quality, specifically mention, for example, the predominantly used waiting list control groups and the lack of blinding of patients to group membership, which can lead to an overestimation of the effect (Goldberg et al., 2023). In addition to these limitations, the implementation of telemedicine and DHIs in standard care poses further challenges.

### 2.3 Implementation

The implementation of telemedicine and DHIs requires careful consideration of the needs and abilities of the target group. Customised training for therapists and patients as well as a user-friendly design of the interventions are crucial for successful integration into mental health care (Shafran et al., 2024). The majority of the population tends to be very accepting of DHIs and see them, for example, as an additional and innovative resource for promoting mental health (Grist et al., 2017; Beames et al., 2023). Especially young people are also generally tech savvy (Feierabend et al., 2021). Nevertheless, there are various barriers to the use of DHIs as with any other application of a method, the consideration of patient rights under the GDPR (General Data Protection Regulation) is decisive (at least in European Countries) and therefore not a new finding. citeborghouts2021barriers, graham2020implementation: Stigma associated with mental health and help-seeking, preference for traditional face-to-face care, concerns about confidentiality, privacy, discomfort with technology or inability to use it, complexity of application, mobile device compatibility issues, low digital literacy, limited research findings, organisational barriers (e.g., scheduling issues), no available technical support, lack of cultural and ethnic diversity, financial costs (e.g., reimbursement, start-up costs), low personalisation, severe mental health issues, negative practitioner attitudes towards technology and resistance to change, and perceived negative impact on consumer safety (Graham et al., 2020). Concerns about AI-guided CBT include the perceived lack of human connection, which some young people feel is important for effective mental health support (Egan et al., 2024). While rates of use in youth are varied, one United States-based study suggested that only 19 % of youth have tried a mental health app with another study from South Africa highlighting the mixed view of youth (Poll et al., 2023). Therapists, in particular, often feel more apprehensive about using DHIs than their clients and face challenges in adapting to new, less conventional methods that disrupt their usual workflow, placing them in the role of gatekeepers (Cowan et al., 2019).

In contrast, the following facilitating factors were discussed (Borghouts et al., 2021; Graham et al., 2020): Advances in technology and quality of care, guidance from a healthcare professional, improvement in therapeutic relationship/relationship, social support through online forums or groups, improved insight into health and feeling in control of own health, peer counselling, ease of use and convenience, availability of online resources and SMS reminders, consumer-centric features, availability of (software) developers, technical equipment and resources for implementation, liaisons to bridge cultural gaps, strong organisational leadership, standardised measures, maximum security, trust in and credibility of the DHI, and lower cost or cost analysis. These factors also influence the frequency of use of self-help services in particular (Borghouts et al., 2021).

Erku et al. (Erku et al., 2023) analysed 65 articles on the implementation of digital technology in primary care. In their conclusion, they summarise the above-mentioned factors necessary for successful implementation as a “well-functioning digital ecosystem – with adaptable, interoperable digital tools, robust information and communication technology foundations and an enabling environment” (p. 1). Erku et al. (2023) also emphasise the relevance of digital skills in facilities with motivated staff and adequate funding for a higher acceptance of eHealth technologies, which leads to improved, coordinated service delivery, and higher patient satisfaction (Erku et al., 2023).

#### 2.3.1 Recommendation for the safe and transparent integration of digital technology into treatment/care

The integration of digital health interventions in mental health care requires careful consideration of several key factors. Firstly, increased evidence is essential, long-term consequences of interventions, and in at-risk populations with limited infrastructure (Seiferth et al., 2023). Research should specifically aim to develop evidence-based approaches and compare new approaches to digital placebo as well as existing services. Secondly, it is crucial to ensure transparency, security, and data protection for all user groups, including e.g., children, parents, elderly participants, therapists, health care providers and teachers. Education about the handling of personal data and security measures should be an integral part of any digital health intervention. This also includes understanding AI, which is often portrayed in the media as a threat that cannot be controlled. This is not only unhelpful and fuels irrational fears, but also prevents the responsible integration of digital support into healthcare. Thirdly, clear guidance should be created to answer the question "What can I use in good conscience?” and provide information on where the generated data flows. Guidelines such as the mindApps.org searchable database (Lagan et al., 2021), the NICE guidelines, and information platforms for users (such as https://mhad.science) can serve as a guide here.

Nevertheless, the methodological requirements should be increased to ensure appropriate quality and effectiveness, comparable to the standards that apply to medical treatments. Finally, research into the side effects of digital health interventions remains inadequate (Schulte et al., 2024; Bear et al., 2022). Here, too, the study situation is poor, but various authors unanimously recommend at least the observation of unintentional effects during the use of DHI (Ziebland et al., 2021; Schuez and Urban, 2020). In addition, the development of a comprehensive understanding of the potential risks is recommended to ensure the safety and effectiveness of digital interventions in child and adolescent psychiatry.

## 3 Future perspectives

In the following, we describe three potential future cases, making use of applied digital health technology, embedded in a regular health service system. Firstly, we focus on the near future, illustrating an ideal scenario for the integration of current technological advancements into mental health care systems. The other scenarios contrast an optimal vision with a more negative, futuristic outlook on how technological developments might evolve within our healthcare systems. Although these scenarios are somewhat visionary, our goal is to highlight the potential challenges and benefits once current emerging developments are fully realised.

### 3.1 Year 2030

In 2030, Alex, a 34-yearold AI ethics consultant, begins experiencing mental health challenges, including sleep disturbances, reduced motivation, and social withdrawal. His AI companion, K-AI, detects concerning changes through digital phenotyping, analyzing data from Alex's smart devices to identify patterns in his biological, psychological, and social behaviours. While K-AI can simulate therapy and suggest interventions, it lacks human empathy and cannot replace professional care (Carlbring et al., 2023). Initially skeptical, Alex feels both validated and uneasy when K-AI mirrors his unacknowledged emotions. After a motivational discussion (Mercado et al., 2023), Alex agrees to seek professional help and schedules an appointment at a mental well-being clinic.

At the clinic, Alex meets Dr. Vega, a specialist in digital therapeutics, who reviews the real-time data collected by K-AI. This data offers a dynamic and comprehensive view of Alex's mental health, revealing patterns that go beyond traditional diagnostic methods (Rauseo-Ricupero et al., 2021). Dr. Vega collaborates with Alex, ensuring he remains actively involved in his care rather than a passive recipient. Using AI for a second opinion to enhance diagnostic accuracy (Chekroud et al., 2021), she develops a personalised and adaptive treatment plan. This plan integrates digital interventions such as mental health apps, virtual reality exposure therapy (VRET) (Ford et al., 2023), and just-in-time adaptive interventions (JITAI), which deliver targeted support based on real-time analytics (Bögemann et al., 2023). Alongside these, traditional psychotherapy sessions help provide the human connection that technology alone cannot offer.

As Alex progresses, K-AI continuously monitors his mental state, providing tailored interventions and acting as an early warning system for potential relapses (Fried et al., 2023). When signs of increased stress or anxiety arise, K-AI suggests behavioural exercises, initially in a virtual reality setting before transitioning to real-world applications. While technology enhances his treatment, it is Dr. Vega's expertise and human insight that ensure interventions remain personalised and emotionally grounded.

Despite the sophisticated support system, Alex still faces challenges, including periods of heightened stress and fluctuations in mood. During these times, Dr. Vega's role becomes even more critical, as she offers guidance, reassurance, and a space for reflection that technology alone cannot replicate. Over time, the treatment evolves based on Alex's progress, fine-tuning interventions to match his needs. To maintain his well-being, Alex and Dr. Vega establish a long-term strategy, incorporating continuous monitoring, self-care practices, and clear relapse prevention measures.

By blending AI-driven support with human expertise, Alex's treatment represents a future where digital technology enhances mental healthcare while preserving the essential role of human connection. The integration of JITAI, continuous K-AI support, and immersive VRET enhances Alex's ability to manage his mental health. The treatment evolves dynamically, adapting to real-time data through digital phenotyping. When Alex experiences stress or anxiety, K-AI guides him in behavioural experiments, first in virtual reality and later in real-life situations.

Despite this advanced system, Alex faces common mental health challenges, such as stress and mood fluctuations. While technology quickly adapts, Dr. Vega's human insight ensures interventions remain trusted and effective. Through regular consultations, she provides essential empathy and guidance that AI cannot replicate.

As Alex improves, he and Dr. Vega recognize the need for ongoing support. K-AI facilitates continuous monitoring as an early warning system for relapse (Fried et al., 2023). Dr. Vega helps Alex develop a relapse prevention plan, incorporating stress management, coping strategies, and clear guidelines on seeking further support. K-AI remains central to this plan, delivering timely interventions and self-care reminders (Chekroud et al., 2021).

**Summary**. This vignette presents a near-future mental health case set in 2030. Alex, an AI ethics consultant, faces mental health challenges, prompting his AI companion, K-AI, to detect concerning patterns and suggest professional help. His treatment combines human expertise with AI-driven interventions like digital phenotyping, JITAI, and virtual reality therapy, creating a personalised plan that balances technology and human support for long-term well-being.

This case highlights current trends and the ideal role of technology in mental health care. With AI-driven advancements evolving at an exponential pace, the possibilities for implementation continue to expand. The following scenario introduces Beta, a character in a more futuristic setting, depicting how these technologies might shape mental health care. Given the unpredictable speed of innovation, no specific timeframe is assigned to this scenario.

### 3.2 Some time later

#### 3.2.1 A: beta's journey: navigating social anxiety in a technologically advanced world

Several years later, Beta finds herself at a crossroads with her social anxiety disorder. Unlike many of her peers, her parents chose not to use gene modification techniques like CRISPR-Cas9, believing in the value of natural genetic diversity (Cong et al., 2013). Instead, they opted for a brain implant designed to support cognitive processes and emotional regulation. The implant, a product of advanced neurotechnology, can predict feelings and behaviours up to five minutes in advance, providing real-time cognitive support. It detects negative thought patterns and introduces alternative perspectives in anxiety-inducing situations, helping Beta challenge automatic negative assumptions. For example, before a holographic meeting, it prompts her to consider positive outcomes and recall past successes. The implant also predicts emotional states, suggesting coping strategies or calming stimuli to manage anxiety and build confidence. Its sleep assistance feature helps Beta process racing thoughts for more restful sleep, improving her overall mental health. Though Beta relies on the implant, she also develops her own coping strategies, like mindfulness. As she continues therapy with the implant, she reflects on the complex relationship between technology and mental health, sometimes questioning authenticity in a world where thoughts can be influenced. These reflections lead to meaningful discussions with family and friends, deepening her understanding of her experience in this technologically advanced world. Looking at the broader landscape, Beta's experience is part of a revolution in mental health. Integration of advanced technology with traditional therapeutic approaches has improved mental health outcomes worldwide. Significant breakthroughs include gutbrain microbiome research, leading to personalised probiotic treatments that alleviate symptoms of mental health disorders like depression and anxiety. These treatments, delivered through engineered foods or targeted therapies, optimise the gut-brain axis, producing neurotransmitters and immune modulators that impact mood and cognition. Advancements also include AI-driven personalised mental health programmes using vast anonymised data to create tailored interventions. By analyzing behaviour patterns and physiological responses, these AI systems predict mental health fluctuations and suggest preemptive measures. Virtual and augmented reality technologies have evolved to create immersive therapeutic environments, allowing individuals to confront phobias, practise social skills, or experience calming scenarios in safe settings, enhancing the effectiveness of exposure therapy and skills training.

Community support has taken on new dimensions with the advent of global empathy networks. These networks use advanced brain-computer interfaces to allow individuals to share emotional experiences in a controlled, consensual manner. This has led to unprecedented levels of understanding and support among diverse groups of people, breaking down social barriers and reducing stigma around mental health issues. Education about mental health is now a cornerstone of global curricula, with children learning about emotional intelligence, cognitive biases, and well-being strategies from an early age, creating a society more attuned to mental health needs. There's also a renewed appreciation for nature's impact on well-being. Urban areas have been redesigned to incorporate extensive green spaces, recognising the powerful effect of nature on mental health. “Forest bathing” and nature-based therapies have become standard prescriptions for stress relief. The holistic approach to mental health in 2100 acknowledges the interconnectedness of mind, body, and environment, leading to more comprehensive treatments and improving quality of life for billions. As Beta continues her journey, she does so in a world that understands mental health challenges better and is equipped with unprecedented tools and support systems. While the path to optimal mental health remains personal, advancements have made that journey more supported and achievable than ever before. Beta's personal data is managed securely through advanced privacy-preserving technologies, ensuring that her mental health information is only accessible to authorised healthcare providers. She retains control over who can access her data, with transparent options for sharing based on her treatment needs and consent. Data is processed in a secure and fair manner. This responsible approach fosters trust, allowing Beta to benefit from personalised care while safeguarding her privacy and autonomy.

**Summary**. Beta's journey with social anxiety disorder highlights the transformative potential of advanced technology in mental health care. Opting for a brain implant rather than gene modification, Beta benefits from a device that provides real-time cognitive support, predicting emotions and behaviours, helping her manage anxiety, and encouraging positive thinking in triggering situations. With features such as sleep assistance and strategies for building confidence, the implant aids her in coping with her condition, though she also integrates mindfulness practices for balance. As she reflects on the influence of technology on her thoughts and well-being, Beta engages in meaningful discussions that deepen her understanding of her mental health journey.

#### 3.2.2 B: omega's journey: navigating mental health in a technologically overrun world

Several years later, Omega finds herself struggling with mental health issues in a world dominated by technology. Despite the promise of advanced interventions, Omega has become increasingly wary of the tools available. Various solutions, such as digital phenotyping, brain implants, and AI-driven therapies, were marketed as revolutionising mental health care, but many of them fall short of their promises. Omega's experience with these technologies has been less than ideal. For example, an AI-powered mental health app promises to improve mood based on biometric data, yet the app frequently misinterprets her emotional state, leading to mismatched interventions. It suggests irrelevant exercises or fails to provide the necessary support during emotional crises, causing Omega to feel more isolated and misunderstood. Despite the app's claims of “personalisation,” it often feels like a generic solution for a complex issue. Similarly, Omega had high hopes for a brain implant designed to assist with emotional regulation. Initially, the implant seemed promising, offering real-time support based on her emotional state. However, it was soon clear that the device was not secure. It transmitted data to third-party companies without full transparency, raising concerns about her privacy. The algorithm behind the implant was based on insufficient data, causing it to malfunction during times of heightened stress, leaving Omega to rely on her own coping mechanisms instead. The rise of digital phenotyping, which collects vast amounts of personal data to predict mental health trends, further fuels Omega's distrust. The data is often misused by companies focused on profit rather than wellbeing. With no clear regulations in place, Omega's personal information is frequently sold to advertisers or used to create interventions that feel invasive, rather than supportive. The free-market nature of these technologies means that they are often prioritised over clinician-led interventions, despite being poorly validated. The lack of evidence-based efficacy and the absence of regulation have resulted in a flood of low-quality, non-trustworthy solutions. These technologies are designed to appeal to consumers but lack the clinical oversight needed to ensure safety and effectiveness. Omega has yet to find a solution that feels both secure and genuinely helpful. The most troubling aspect of her experience is the constant push to adopt new technologies that promise easy fixes, bypassing clinical wisdom in favour of market-driven trends. These solutions, while promising, often fall short in quality, usability, and safety. Omega feels increasingly caught between her need for effective mental health support and the overwhelming presence of subpar technological interventions that seem to be more focused on profit than people's well-being. In a world where personal data is the currency of digital health, Omega struggles to trust any of the technologies available. The lack of regulation and oversight leaves her with a sense of vulnerability, as her data is used for purposes far beyond her control or consent. What started as a hopeful journey into the future of mental health care has now turned into a cautious exploration of its risks and limitations.

In contrast to Omega, Beta's experience is part of a broader revolution in mental health, where the integration of technology and traditional therapies leads to improved outcomes globally. Advances like personalised probiotic treatments, AI-driven mental health programmes, and virtual reality therapy provide targeted interventions that enhance treatment effectiveness. Furthermore, community support is strengthened through global empathy networks and educational initiatives, while urban redesigns prioritise nature for stress relief. In this future, mental health is approached holistically, addressing the mind-body-environment connection, while ensuring privacy and autonomy through secure data management. These developments create a world where mental health challenges are better understood, supported, and addressed, offering individuals like Beta unprecedented resources for their journey to well-being. However, Omega's case indicates numerous dangers and concerns, clinicians as well as researchers must be aware of and take action.

## 4 Recommendations for integrating technology in mental health care

Technological progress opens up new opportunities and challenges for psychiatry and psychotherapy. But technology is neither a panacea nor exempt from the rigor required to enter the clinical sphere. Perhaps the single greatest threat to technological progress is not the technology itself but the dangers of technology exceptionalism: exempting innovation from the highest methodological quality, data security, and transparency. A commitment to comprehensive and rigorous information is essential and should accompany the implementation of digital interventions in practice. Herein, we provided two views on a potential technology-aided future - near- and distant-term. Obviously, predicting in particular a distant future is merely speculative in many ways. For example, it is unclear to which degree humans will be augmented by technology, to which degree they will be supported and surrounded by AI and robotics, and to which degree they will spend time in virtual or augmented worlds and nets. Independent of that, next, we will distill **TEQUILA**: Necessities and challenges to create a beneficial future of digital health technologies. To build trustworthy digital health tools for mental well-being, several key factors must be addressed to ensure efficacy, safety, and user confidence. This acronym serves as a foundation for developing future recommendations on integrating technology into mental health care, drawing on the author's expertise in the field. The seven parameters that build **TEQUILA** were selected based on a) their potential to improve methodology in the field, b) their proven value in former studies, and c) their theoretical significance.

**Trust**: Data and algorithm security and privacy as well as transparency in and fairness of algorithms and AI-driven decision-making are paramount. For example, current AI is hardly robust against “adversarial” attacks by other AI and often not trained to cope with miss-usage. It is often further not yet fully ready for atypical patterns not encountered during training. Yet in 2024, trust remains aloof with as many as one in five mental health apps still with privacy concerns and regulators bringing cases of brazen breaches of patient trust against large companies in the space (Iwaya et al., 2023). Yet, trust is obtainable and the current challenges in the space are less due to technical concerns than a commitment to preserving privacy from companies in the space (US FTC, 2024b). A future world where trust is high in digital health companies is possible today.

**Evidence**: First, robust evidence-based design is essential, ensuring that interventions, sensing tools, VR/AR applications, and holographic technologies are grounded in solid clinical research and demonstrate measurable benefits for mental health. The majority of digital mental health technology studies lack adequate control groups and so do not account for a digital placebo effect. The impact of relying on uncontrolled pilot studies to assess intervention efficacy is best seen in schizophrenia, where the field's leadership in higher quality research studies has shown many negative results when a digital control group is utilised (Torous et al., 2024; Bucci et al., 2024). Such negative results ensure that research can steer in the right direction and scientific effort is most productive.

**Quality**: Ongoing regulatory oversight and continuous evaluation of these tools, including long-term user feedback and clinical validation, will help maintain their reliability, ensuring they remain safe and effective as technology evolves. While there is a natural tension between regulation and innovation, relying on high quality evidence to guide decisions around policy offers one solution. Given the nature of digital health tools intended for use outside of clinical studies and in the real world environment, demanding continuous feedback and evaluation data is imperative. Regulators need access to real-time results of real-world performance, and thankfully this is easy to share given the digital nature of these interventions.

**Usability**: Ensuring usability and accessibility is another crucial component, as tools must be intuitive, inclusive, and adaptable to diverse populations, including those with varying levels of digital literacy and different cultural or socioeconomic backgrounds. Often the populations with the lowest levels of digital literacy are those most in need of care. A recent appreciation of the need for resources and action in this space reflects a growing trend to consider usability in a broader sense than simply engagement (Robinson et al., 2024). These broader efforts addressing the social determinants of health will likely find greater success than less fruitful efforts to improve usability through technical solutions like gamification or chatbots (Koivisto and Hamari, 2019). In digital approaches, practicability often adds to usability, such as efficient AI algorithms that are conservative on battery consumption and do not require too much additional installation of software, update thereof or additional hardware as well as short training and processing times.

**Table:** 

TEQUILA	Recommendation
Trust	Data security, privacy, and transparency in AI are crucial, as current AI faces issues with attacks, misuse, and privacy concerns in mental health apps
Evidence	Robust evidence-based design is crucial to ensure that mental health technologies are grounded in solid clinical research, leading to effective interventions with measurable benefits
Quality	Ongoing regulatory oversight and continuous evaluation, including long-term user feedback and clinical validation, ensure that digital health tools remain reliable, safe, and effective as technology evolves.
Usability	Ensuring ease of use and accessibility is crucial, particularly for populations with low digital literacy, and addressing social health determinants is more effective than relying on technical solutions like gamification.”
Interest	Interventions must prioritise end-users' interests, with ethical considerations around autonomy and consent. Aligning these interests involves addressing who funds, controls, and uses data, and incorporating peer support to ensure inclusivity and relevance in digital mental health tools
Liability	Legal considerations, especially around liability, are critical before deploying AI-driven mental health services. Responsibility for diagnoses and interventions lies across data, algorithms, service providers, and overseeing bodies, requiring careful attention.
Accreditation	Due to the tendency of AI models to hallucinate and their error-prone nature, human oversight and accreditation by relevant boards for ‘Dr. AI’ are essential to ensure compliance with the TEQUILA principles

**Interest**: All interventions must primarily serve the interest of the end-users: Ethical considerations around user autonomy and consent are critical. Today some endusers are skeptical of digital health solutions (Sawrikar and Mote, 2022; Daele et al., 2022) while others see the promise (Buck et al., 2024), but a compelling universal use case implemented into routine digital mental health remains aloof. Aligning end-users' interests requires considering issues around who pays for the development and upkeep of digital health tools, who has access to the data necessary for improving these tools, and how the data may be used to expand or limit access to other forms of care. The expanding role of peer support and people with lived experience in digital mental health provides the best foundation to ensure that these interests are built into the newest generation of tools.

**Liability**: Beyond scientific and technological aspects, legal questions will need to be answered before services described above can be provided. Among the challenging questions in this respect, one finds liability - a topic that accompanies largely to fully autonomous AI in many other use cases such as autonomous driving. The responsibility that lies in diagnoses and interventions provided by AI cannot be underestimated, but the sources of liability will be rich lying in data, algorithms, service providers, and those overlooking these.

**Accreditation**: Given hallucination of today's large AI models and the nature of statistical machine learning to be prone to errors, it becomes clear that not only human oversight will remain needed, but also some general form of accreditation for “Dr AI” by suited boards. These will have to assure adherence to the TEQUILA principles as named above.

In summary, the TEQUILA framework emphasises essential principles for ensuring the trust, quality, and effectiveness of digital mental health technologies. It highlights the importance of secure data handling, evidence-based interventions, continuous regulatory oversight, user-centred design, and ethical considerations, alongside addressing legal liabilities and the need for accreditation to ensure reliable and responsible AI-driven care. Since AI is increasingly used by community members and mental health professionals (Cross et al., 2024), we aimed to highlight benefits and potentials harms for the integrations of innovative technology into mental health care.

To conclude, with the current rapid process in AI and technology a new era of digital psychology has begun that opens up entirely new horizons of possible realtime diagnosis, summarisation of 24/7 monitoring for therapists, but also largely autonomous interventions. It will be of crucial importance to follow-up rapidly with assuring the quality, robustness, and safety of these to exploit their full potential.

## Declaration of competing interest

The authors declare that there are no conflicts of interest regarding the publication of this paper: *Digital Interventions in Mental Health: An Overview and Future Perspectives.*
